# Proceedings of De Witt County Medical Society

**Published:** 1865-08

**Authors:** 


### PROCEEDINGS OF THE DEWITT CO. MEDICAL SOCIETY.

The Society met in quarterly session at the office of Dr. Madden, in Clinton, on Monday, the 3d day of July. The President, Dr. Tyler, in the chair.

The minutes of the last meeting were read and approved. Drs. Adams and Madden, the essayists, being unprepared, were requested to read their essays at the next meeting. Dr.

Davis, of Wapello, exhibited to the Society a specimen of monstrosity, giving a full history of the case, for which he received the thanks of the Society.

Dr. Adams made a verbal report of three cases of cerebro-spinal meningitis, which led to a discussion upon the pathology and treatment of this disease, in which most of those present partook.

Dr. Lewis reported an interesting case of diphtheria of a child two years old, giving treatment and favorable result, which called up another animated discussion, in which most of the members participated.

Gunshot Wounds was chosen as the subject for discussion at the next meeting.

On motion, it was ordered that the secretary furnish a copy of these proceedings to the *Chicago Medical Journal* and *Chicago Medical Examiner* for publication.

On motion, the Society then adjourned to meet in semi annual session in Clinton, on the 1st Monday in October, at 10 o'clock, A. M.

At this meeting we had the extreme pleasure of taking by the hand our esteemed friend and fellow member, Dr. John Wright, late surgeon of the 107th Reg. Ill. Vol. Inf., who has just returned from the field, after serving the country creditably for three years. CHRISTOPHER GOODBRAKE, M.D., *Sec.*

